# NFATc2 mediates epigenetic modification of dendritic cell cytokine and chemokine responses to dectin-1 stimulation

**DOI:** 10.1093/nar/gku1369

**Published:** 2014-12-30

**Authors:** Hong-Bing Yu, Marina Yurieva, Akhila Balachander, Ivy Foo, Xiangrong Leong, Teresa Zelante, Francesca Zolezzi, Michael Poidinger, Paola Ricciardi-Castagnoli

**Affiliations:** Singapore Immunology Network (SIgN), Agency for Science, Technology and Research (A*Star), Biopolis, Singapore

## Abstract

The transcription factor NFATc2 regulates dendritic cell (DC) responses to microbial stimulation through the C-type lectin receptor dectin-1. But the genetic targets of NFATc2 and their effects on DC function remain largely unknown. Therefore we used ChIP-seq to conduct genome-wide mapping of NFATc2 target sites in dectin-1-activated DCs. By combining binding-site data with a comprehensive gene expression profile, we found that NFATc2 occupancy regulates the expression of a subset of dectin-1-activated genes. Surprisingly, NFATc2 targeted an extensive range of DC-derived cytokines and chemokines, including regulatory cytokines such as IL2, IL23a and IL12b. Furthermore, we demonstrated that NFATc2 binding is required to induce the histone 3 lysine 4 trimethylation (H3K4me3) epigenetic mark, which is associated with enhanced gene expression. Together, these data show that the transcription factor NFATc2 mediates epigenetic modification of DC cytokine and chemokine genes leading to activation of their expression.

## INTRODUCTION

Dendritic cells (DCs) are antigen presenting cells that express Toll like receptors (TLRs) and C-type lectin receptors (CLRs) that mediate the detection of microbial pathogens and stimulate DCs to induce appropriate immune responses against bacteria and fungi. Dectin-1 belongs to the CLR family of receptors and mediates DCs recognition of β-glucan carbohydrates, which are produced by various species of fungi including *Candida albicans* and *Aspergillus fumigatus* (1). Upon ligation by β-glucan, dectin-1 undergoes Src kinase-mediated phosphorylation of the immunoreceptor tyrosine-based activation motif (ITAM), docking of Syk and activation of the phospholipase C (PLC)-gamma2-dependent Ca^2+^-calcineurin/nuclear factor of activated T-cells (NFAT) signaling pathway (2,3). Alternatively, DCs stimulation through dectin-1 can also lead to Syk/Raf1-mediated activation of both canonical and non-canonical NF-kB signaling pathways (4–8). While these upstream components of Ca^2+^-calcineurin/NFAT signaling in DCs are well documented, the downstream signaling events in the Ca^2+^-calcineurin/NFAT signaling pathway remain poorly defined, and their potential impact on DCs function is currently unclear.

NFATc1/2 have already been shown to exert potent effects on host immune responses. In myeloid lineage cells, including DCs, microbial activation of NFATc1/2 critically regulates the expression of several key cytokines including IL2 (9–13), as reviewed in (14). NFATc2-dependent IL-2 production by DCs has been shown to play a key role in the priming and activation of lymphocyte populations including regulatory T cells (Treg) (15), and modifies NK cell function *in vivo* (16–19), hence this transcription factor regulates several key pathways that shape the host response to invading pathogens.

In contrast to NF-κB signaling, which regulates the immune response to infection in both vertebrates and invertebrate species, the first appearance of NFAT family members around 500 million years ago coincided with the appearance of vertebrates and the development of adaptive immunity (20). This suggests that Ca^2+^-calcineurin-NFAT signaling may have evolved to overcome the unique challenges of increasing organismal complexity, acting as a mechanism to regulate antigen-specific immune responses in order to protect against autoimmune pathologies and prevent chronic inflammation. This concept is supported by the ability of NFAT family members to modulate leukocyte cytokine expression during both innate and adaptive immune responses, indicating a key role for the NFAT transcription factors in controlling the balance of inflammation versus regulation in host tissues. Indeed, NFATc2 in particular has previously been implicated in cell fate regulation and apoptosis induction in lipopolysaccharides (LPS)-stimulated DCs, which is an essential mechanism for limiting inflammatory responses to microbial pathogens (21). It is surprising therefore that so few gene targets of NFAT have been identified to date, which has restricted the therapeutic manipulation of this pathway for clinical benefit in human inflammatory diseases.

The transcription factor NFAT was first discovered over 25 years ago and was initially identified as a regulator of T cell activation genes (22), hence NFAT has, until recently, been thought to modify immune responses only within the lymphocyte compartment. Indeed, the potent Ca^+^-calcineurin/NFAT inhibitor drugs CsA and FK506 are widely used to prevent allograft rejection in human patients, and the efficacy of these drugs is thought to depend on inhibition of IL-2 synthesis by graft-derived and patient T-cells. However, experiments in rodent models have demonstrated that inhibition of Ca^2+^-calcineurin/NFAT signaling can also lead to decreased cytokine production by DCs during fungal infection (23), suggesting that the effects of NFAT inhibition on non-lymphoid cell types may contribute to the therapeutic efficacy of these drugs. Indeed, we and others have previously shown that Ca^2+^-calcineurin/NFAT signaling is a common feature of myeloid cells, and of DCs in particular (14,21). However the impact of Ca^2+^-calcineurin-NFAT signaling on DC function and the influence of this pathway on host protection against pathogens is currently unknown.

In order to assess the role played by NFATc2 in shaping DC responses to microbial antigens we conducted genome-wide mapping of NFATc2 binding sites in activated murine DCs. To achieve this, we stimulated the well-characterized long-term murine splenic DC line (D1) (24) with the dectin-1 ligand curdlan (21,25–28), either in the presence or absence of NFAT inhibitors, and used unbiased ChIP-seq technology to map NFATc2 binding sites and profile ligand/inhibitor-induced changes in gene expression.

By integrating ChIP-seq data with gene expression profiles, we demonstrate that NFATc2 occupancy is responsible for curdlan-mediated gene activation, and we present the first exhaustive list of NFATc2-regulated genes in DCs. The NFATc2 target genes incorporated an extensive range of DC-derived cytokines and chemokines, including regulatory cytokines such as IL2, IL23a and IL12b. In addition, our data reveal that NFATc2 recruitment is essential for the induction of the H3K4me3 epigenetic mark which is associated with enhanced gene expression in curdlan-stimulated DCs, thus indicating that this epigenetic modification is associated with NFATc2 binding.

## MATERIALS AND METHODS

### Cell culture

The murine DC line (D1) was cultured as previously described (9,24) In brief, cells were maintained in Iscove's modified Dulbecco's medium (IMDM; Sigma, St. Louis, MO, USA) containing 10% heat-inactivated fetal bovine serum (Gibco-BRL, Gaithersburg, MD, USA), 100-IU/ml penicillin, 100-μg/ml streptomycin, 2-mM L-glutamine (all from Sigma) and 50-μM β-mercaptoethanol. The cultures were supplemented with R1 supernatant from NIH3T3 fibroblasts transfected to express GM-CSF (30% of total DC culture volume). The D1 cells were infected with lentiviral vectors and DCs displaying low-level internal ribosomal entry site green fluorescent protein (IRES-GFP) expression, were sorted and expanded for use in chromatin immunoprecipitation (ChIP) experiments (29).

Bone marrow cells from 8–12-week-old C57BL/6 mice were cultured in IMDM (containing 10% heat-inactivated fetal calf serum (FCS) (Life Technologies), penicillin 100 U/ml, streptomycin 100 μg/ml and 10% B16-GMCSF growth supernatant for 7 days. On day 8, mature bone marrow-derived DCs (BMDCs) were stimulated with 10-μg/ml curdlan in the absence or presence of 200-ng/ml FK506 for experiments.

### Immunofluorescence

D1 cells were seeded into 8 well μ-slides (Ibidi) and rested for 2 h before stimulation with curdlan (10 μg/ml) for various times prior to fixation in 2% paraformaldehyde (PFA). The cells were then re-suspended in permeabilization buffer (0.1% saponin in D-PBS containing 5-mg/ml bovine serum albumin and 0.2% gelatin), immunolabeled with antibodies against NFATc2 (Thermo Scientific) or V5 tag (Invitrogen) and finally counter-stained with 4', 6-diamidino-2-phenylindole (DAPI) and anti-Alexa488 antibody (Life technologies).

### Chromatin immunoprecipitation

ChIP assays were conducted using D1 cells that were stably expressing NFATc2 with a C-terminal V5 tag (29,30). Briefly, cells were treated with 1% formaldehyde for 10 min at room temperature to cross-link transcription factors to chromatin. The formaldehyde was then inactivated by addition of 125-mM glycine. Sonicated chromatin extracts containing DNA fragments with an average size of 500 bp were immunoprecipitated using 6-μg mouse IgG (Santa Cruz Biotechnology) or V5 control monoclonal antibodies (Invitrogen) with pre-blocked protein G-Sepharose beads and then incubated overnight at 4°C. The following day, the chromatin-protein-antibody-bead complexes were eluted and the ChIP DNA was extracted. For all ChIP experiments, quantitative polymerase chain reaction (PCR) analyses were performed in real time using the ABI PRISM 7900 sequence detection system with SYBR Green master mix (Promega). Relative occupancy values were calculated by determining the immunoprecipitation efficiency (quantity of immunoprecipitated DNA expressed as a ratio of total input) for a given region of interest compared with the control region.

### ChIP-seq identification of NFATc2 binding sites

ChIP-seq libraries were prepared from a total of 10 or 20-ng starting DNA using the Illumina TruSeq DNA version 2 kit according to the manufacturer's protocol, with the following modifications; 30X adaptor dilution and 18 PCR cycles of amplification. Batches of five to eight samples were sequenced as a multiplex in individual lanes of an Illumina HiSeq 2000 apparatus using a single-end run (1×51) together with the SBS kit v3. Reads were mapped to the mouse genome (version mm9) with bowtie 1 (31) using default parameters. The MACS 1.4.1 program was then used with default settings to detect peaks of ChIP enrichment (32) relative to a control data set that was generated using input material from the same cell line.

### Luciferase reporter assays

The promoter regions including the Zfp206-binding sites for IL2, IL12b and IL23a were cloned into pGL3-basic vector (Promega). A dual luciferase system (Promega, Madison, WI, USA) was used. For the luciferase assay in 293T cells, 4 × 105 cells were seeded into one well of a 24-well plate. After 18 h, 275 ng of luciferase reporter plasmid, 1 ng of plasmid pRL-SV40 and either 1 μg of NFATc2 overexpression vector or empty vector were co-transfected into the cells using Lipofectamine 2000 (Invitrogen). The pRL-SV40 plasmid served as an internal control for normalizing the transfection efficiency. After 40 h of transfection, the 293T cells were lysed, and the luciferase activity was determined with the dual luciferase system (Promega) using a Centro LB960 96-well luminometer (Berthold Technologies).

### Microarray expression analysis

Affymetrix Mouse Gene 1.0ST Arrays were used to profile gene expression in curdlan-stimulated D1 cells (2- or 4-h exposure) that had been treated or not with 200-ng/ml FK506 for the duration of culture. Total RNA was isolated by acid guanidinium thiocyanate-phenol-chloroform extraction (Trizol, Invitrogen) followed by Qiagen RNeasy clean-up procedure. Total RNA integrity was assessed by Agilent Bioanalyzer and RNA Integrity Number (RIN) was >9.1 for all samples. Target ssDNAs were prepared using WT Expression Kits (Ambion/Affymetrix) according to the Affymetrix protocol, then fragmented and hybridized to standard arrays for 17 h at 45°C. The arrays were washed and stained using the fluidics station prior to analysis using a GeneChip Scanner 3000.

### Bioinformatics analysis

Data were normalized using the Robust Multi-Array Average approach (33) and differential gene expression was determined using Limma (34) in R v2.15.2. A Benjamini-Hochberg-corrected *P*-value <0.05 and a cut-off fold-change value of ≥1.5 were applied to identify genes that displayed statistically significant changes in expression.

### Motif analysis

For each library, three groups of 600 randomly selected peaks were created. For each peak, an ∼200-bp genomic sequence within ±100 bp of the peak max was analyzed. For each group of 600 sequences, a motif was generated using default parameters in MEME (35) (*P*-value <0.05) with mode set to ‘One Occurrence per Sequence’ (35,36). Transfac motif database (version 2012.4) was used to obtain the consensus sequence of the NFAT binding motif (ID: V$NFAT_Q6, sequence: NANWGGAAAANN). The NFATc2 co-motif analysis was performed on the 423 peaks found near the genes differentially expressed in D1 cells treated or not with 200-ng/ml FK506 for 2 h. The peak sequences were screened for known motifs in the TRANSFAC database and the results were filtered out using core score cutoff = 1.

### Data access

The data from this study have been submitted to the NCBI Gene Expression Omnibus (GEO) (http://www.ncbi.nlm.nih.gov/geo) under accession numbers GSE59896 and GSE59998.

## RESULTS

### A tagging system for genome-wide mapping of NFATc2 binding sites in DCs

**Figure 1.**
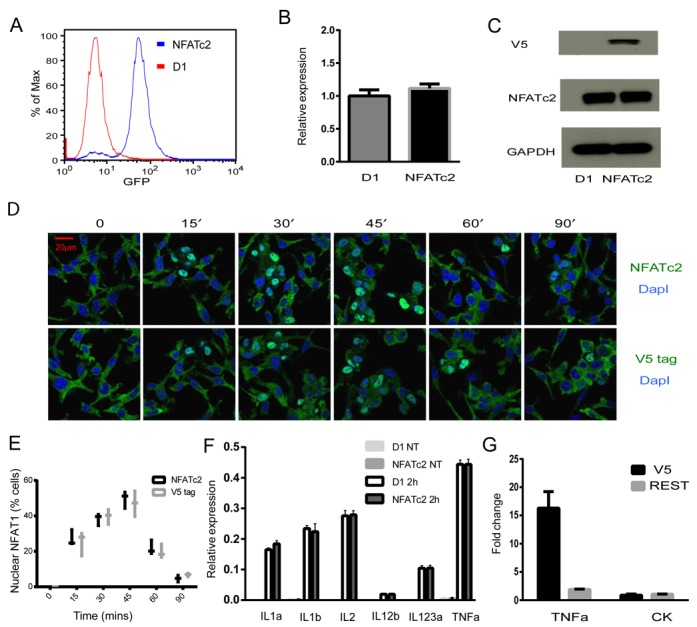
NFATc2-expressing D1 cells can be identified by low-level V5 tag expression. (**A**) Flow cytometry analysis of GFP+ NFATc2-expressing D1 cells. (**B**) Quantitative RT-PCR analysis of NFATc2 mRNA levels in NFATc2-tagged D1 cells. Samples of cDNA were prepared from D1 cells and NFATc2-V5-expressing D1 cells and then interrogated using specific primers for NFATc2 and the housekeeping gene Actb. Data are expressed as fold difference relative to non-transduced D1 cells (bars indicate standard error of the mean of three biological replicates). (**C**) A total of 15-μg protein derived from either control D1 cells or NFATc2-tagged D1 cells was labeled with antibodies against V5, NFATc2 and GAPDH. (**D**) NFATc2 nuclear translocation kinetics was assessed by microscopy in D1 cells after immunolabeling for NFATc2 or V5 tag. (**E**) NFATc2 translocation kinetics.(**F**) Quantitative PCR analysis of cytokine mRNA levels in NFATc2-tagged D1 cells. Samples of cDNA were prepared from curdlan-stimulated D1 cells (2- or 4-h exposure) and then interrogated using specific primers for the house keeping gene Actb and various cytokine genes. (**G**) Chromatin immunoprecipitation of V5-tagged NFATc2 protein was carried out using an anti-V5 antibody (or anti-REST control). ChIP DNA was interrogated by qPCR using primers against the IL2 and TNF promoters. Data are presented as mean ± SD of triplicate experiments.

The NFAT family of transcription factors regulates anti-microbial cytokine responses in myeloid lineage cells, but the mechanisms underpinning NFATc2 signaling in DCs and the influence of this pathway on DC function remain poorly defined. We therefore sought to identify the genome-wide binding targets of NFATc2 in the murine DC cell line D1 using ChIP-coupled high-throughput sequencing (ChIP-seq) to identify physical binding of this transcription factor to specific regions of DNA (37,38). While there are various antibodies against NFATc2 available from commercial sources, none of those tested here were able to yield enrichment of NFATc2 at known target regions of the IL-2 gene promoter (Supplementary Figure S1; (22)). We therefore used an alternative V5 protein tagging approach to conduct the NFATc2 ChIP assays (29,39), together with a GFP reporter gene under the control of IRES to identify transgene-expressing cells (Figure 1A). Quantitative RT-PCR and western blotting indicated that the ratio of tagged transgene expression to endogenous gene expression was less than 1.2:1 (Figure 1B and C). We also confirmed that DC expression of tagged NFATc2 did not significantly alter cytokine production, NFATc2 signaling or DC maturation; in curdlan-stimulated DCs, we observed that both transgenic and endogenous NFATc2 displayed comparable translocation dynamics (Figure 1D and E) and that mRNA expression of IL1A/B, IL2, IL12B/23A, tumor necrosis factor alpha (TNFα) and surface expression of DC maturation markers were not significantly altered by transgene expression (Figure 1F and Supplementary Figure S2). Finally, we used an anti-V5 mAb to confirm recruitment of NFATc2 to its known target site in the TNF promoter region (Figure 1G) (40). Together, these data validated our novel V5 tagging approach/ChIP-seq method of identifying NFATc2 binding sites in murine DCs.

### Global mapping of NFATc2 binding sites in murine DCs

**Figure 2.**
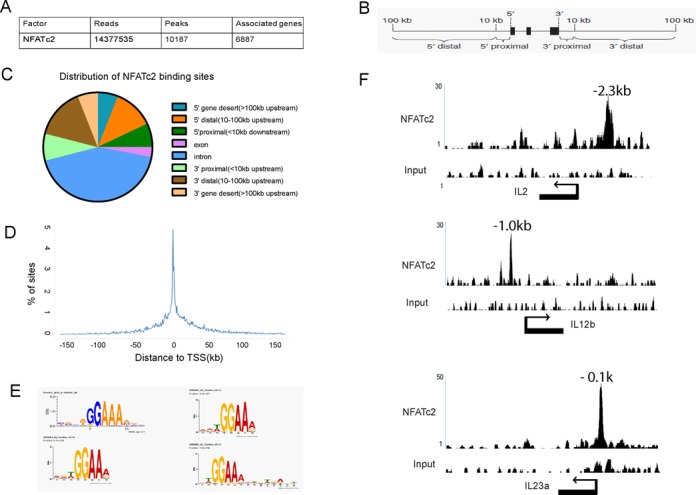
Genome-wide mapping of NFATc2 binding sites by ChIP-seq analysis of murine DCs. (**A**) Table shows the number of uniquely mapped reads, the number of peaks identified and the number of genes associated with the peaks. (**B**) Schematic diagram illustrating how binding site locations were defined; 5′ distal, 5′ proximal, 3′ proximal and 3′ distal sites in the regions 100 kb upstream and 100 kb downstream of the transcriptional unit. (**C**) Pie chart illustrating the distribution of NFATc2 binding sites in relation to genes. (**D**) Line chart showing NFATc2 binding site distribution relative to the nearest TSS. (**E**) *De novo* prediction of the NFATc2 binding motif using MEME software. (**F**) Examples of NFATc2 binding loci within IL2, IL12b and Il23a promoter region were shown.

NFATc2-bound DNA fragments were obtained by ChIP using an anti-V5 antibody and were then subjected to ultra-high-throughput sequencing on the Illumina HiSeq 2000 platform. For NFATc2 ChIP-seq, 1.4 million sequence reads were uniquely mapped to the mouse genome, and a total of 10 187 binding peaks were identified in the proximity of 6887 target genes using the MACS algorithm. The distribution of NFATc2 binding sites was analyzed using a similar annotation to that described previously (gene desert, 5′ distal/upstream promoter, 5′ proximal, intragenic, 3′ proximal, 3′ distal; Figure 2B) (41). Using this approach, we observed that 46% of NFATc2 binding sites were located within a known gene, 7% were situated within a proximal promoter and 12% within a distal promoter (Figure 2C). This profile of binding sites is common among transcription factors (41), and, accordingly, we observed that transcriptional start sites (TSSs) were enriched in the vicinity of NFATc2 binding sites (Figure 2D).

Consistent with ChIP-seq data, we observed NFATc2 enrichment at 12 randomly selected binding peaks that were analyzed by ChIP-PCR (Supplementary Figure S3), and inhibition of NFAT translocation using FK506 reduced the recovery of these regions by ChIP (Supplementary Figure S3), thus confirming the specificity of transcription factor binding to these sites. We therefore proceeded to determine the NFATc2 consensus binding sequence using the MEME algorithm, which generated three distinct binding motifs from three sets of 600 randomly selected peaks (each being located within ±100 bp of a binding peak max). Consistent with reports from other investigators, we identified the NFATc2 binding motif as 5-′GGAAAA-3′ (Figure 2E) (42,43).

### NFATc2 mediates dectin-1 activation of a range of cytokine and chemokine genes in DCs

DCs produce a plethora of cytokines and chemokines that play critical roles in immune regulation. Several key modulatory cytokines have been shown to depend on NFAT family members for their expression, so we hypothesized that at least a subset of DC-derived cytokines and chemokines were likely to depend on NFATc2 for their transcription. We therefore extracted a list of cytokines that were identified by NFATc2 ChIP-seq (Supplementary Table S1), and, consistent with previous findings, we observed that NFATc2 binds to several known target genes including IL-2, TNF, IL-10, GM-CSF, Egr2 and Egr3 (Figure 2F and Supplementary Table S1). In addition, we detected NFATc2 binding to multiple pro-inflammatory cytokine genes including IL1a, IL1b, IL17d and IL22, but also IL12b and IL23a that were previously identified as targets of NF-κB (Figure 2F) (44). We also identified several chemokine genes that were occupied by NFATc2 (Supplementary Table S1). Of these, CXCL2 has previously been shown to be regulated by NFAT in microglia (45), and Cxcl8 expression was reported to depend on NFAT in adenocarcinoma cells (46).

**Figure 3.**
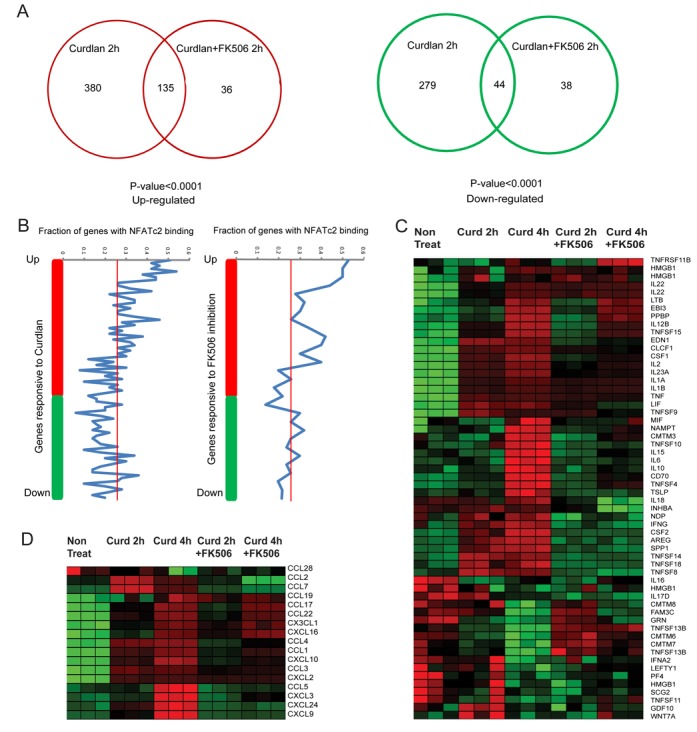
NFATc2 targets a subset of dectin-1-modulated cytokine and chemokine genes. (**A**) Venn diagram displaying the number of genes modulated by dectin-1 and/or by NFAT inhibitor FK506 with curdlan stimulation of murine DCs. The data indicate a statistically significant overlap of NFATc2 target genes with dectin-1-regulated targets. (**B**) Moving average genes in a window of 50 genes that are within 20 kb of NFATc2 ChIP-seq binding site are shown. Significantly differentially expressed genes were identified by comparing DCs stimulated with curdlan for 2 h with either non-treated controls or with DCs co-treated with curdlan and the NFAT inhibitor FK506 for 2 h (*P* < 0.05). (**C**) Heat map displaying cytokine clustering. (**D**) Heat map displaying chemokine clustering.

We next sought to determine which NFAT-responsive genes in DCs were modulated by activation through dectin-1. To do this, we designed a microarray experiment to compare the gene expression profiles of DCs exposed to curdlan for 2 or 4 h, with DCs that were co-treated with curdlan and NFAT inhibitors, and untreated control DCs. Using a cut-off fold-change value of 1.5 (*P* < 0.01), we identified a number of genes that were significantly differently expressed following curdlan treatment and/or by NFAT inhibition (Supplementary Table S2). We also discovered that the majority of NFAT-regulated genes required dectin-1 activation to modulate their expression (Figure 3A). In total, we identified 515 genes whose expression was upregulated by dectin-1 stimulation and 323 whose expression was downregulated. Of these, the expression of 171 genes was upregulated dependent on NFAT, and 82 were downregulated: within this set, 135 genes were common targets for upregulation by both dectin-1 and NFAT, representing 26.2% of the total dectin-1-activated genes, and 78.9% of NFAT-activated genes, suggesting that the NFAT pathway is one of the multiple downstream signaling pathways that can be activated by dectin-1 ligation in DCs. We also detected upregulation of several known NFAT target genes in curdlan-stimulated DCs, including IL2, Egr2 and Egr3 (2).

To further understand how NFATc2 contributes to gene modulation in DCs, we next plotted the fold change in expression level for each gene versus the fraction of those genes that displayed NFATc2 occupancy (Figure 3B). As expected, NFATc2 occupancy was enriched among NFAT-responsive target genes, but we also observed over-representation of NFATc2 binding among the upregulated but not downregulated genes in curdlan-stimulated DCs, suggesting that this transcription factor mediates early activation of target sequences rather than exerting suppressive effects (Figure 3B). Indeed, NFATc2 targets were substantially enriched among the differentially expressed genes in curdlan-stimulated DCs at 2 h, but not at 4 h (data not shown), and heat-map clustering identified several distinct groups of NFATc2-modulated cytokine and chemokine genes whose expression level and sensitivity to NFAT inhibition varied over time (Figure 3C and D). Group 1 cytokines (IL2, IL12b, IL23a, TNFSF15, EDN1, CLCF1 and CSF1) were induced at both 2 and 4 h, and were moderately impaired by NFAT inhibition (Figure 3C). Group 2 cytokines (NDP, IFNγ, CSF2, AREG, SPP1, TNFSF8, TNSF14 and TNFSF18) displayed a similar temporal expression pattern to group 1 but exhibited greater sensitivity to inhibitor treatment (Figure 3C). Finally, group 3 cytokines (IL6, IL10, CD70, TNFSF 4 and TNFSF10) were induced only after 4 h (Figure 3C). Group 1 chemokines (CCL1, CCL2, CCL4, CCL7 and Cxcl10) were significantly induced by NFAT and repressed by the inhibitor at both 2 and 4 h, whereas group 2 chemokines (CCL5, CXCL3, CCL24, CXCL9) were also inhibitor-sensitive but were significantly induced only after 4 h of curdlan stimulation (Figure 3D). Together, these data indicate that NFATc2 is responsible for mediating the early activation of cytokine and chemokine genes in dectin-1-activated murine DCs.

### NFATc2 directly modulates cytokine and chemokine gene expression in DCs via histone modification

**Figure 4.**
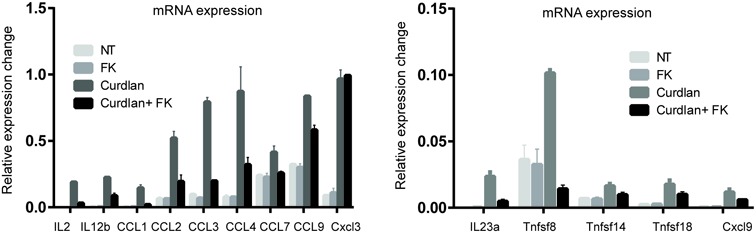
Expression of NFATc2 target genes in mouse BMDCs. Quantitative PCR analysis of cytokine mRNA levels in non-treated (NT), FK506-, Curdlan- and Curdlan+FK506-treated BMDCs. Samples of cDNA were prepared and then interrogated using specific primers for the house keeping gene Actb and various cytokine genes. Data are presented as mean ± SD of triplicate experiments.

We next integrated NFATc2 ChIP-seq data with gene expression data in order to identify the direct targets of NFATc2 regulation (defined as sites within 30 kb of a TSS that displayed both increased NFATc2 binding upon dectin-1 stimulation, and decreased binding in the presence of FK506). Using this approach, we identified a total of 21 cytokine and chemokine genes that were directly regulated by NFATc2 in DCs (Supplementary Table S3). In order to confirm NFATc2 regulation of the cytokine and chemokine genes identified by microarray, we next used qPCR to verify mRNA expression levels and successfully validated expression level changes in 16 of the 21 candidate genes (Supplementary Figure S4). We also measured the expression levels of these cytokine genes in BMDCs to confirm that the results from D1 cells were representative of primary DCs. We found that IL2, IL12b and IL23a gene expression was similarly induced by curdlan stimulation and inhibited by Fk506 treatment (Figure 4). In addition, the other selected NFATc2 target cytokine was also induced and repressed by curdlan and FK506 treatment, respectively, except Cxcl3 (Figure 4).

**Figure 5.**
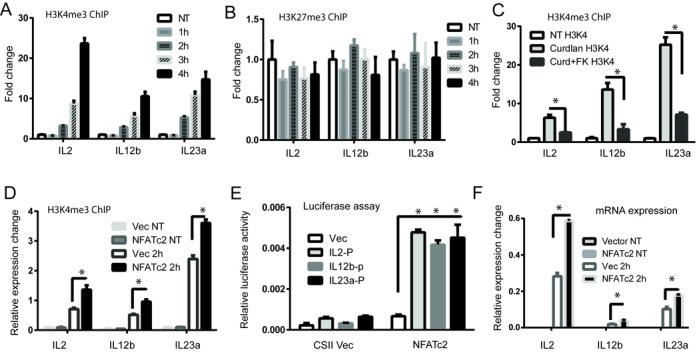
NFATc2 recruitment is associated with enrichment of histone modification H3K4me3 at active genes. (**A**) Chromatin immunoprecipitation was conducted using an anti-H3k4me3 antibody in D1 cells that had been stimulated with curdlan for 1–4 h. (**B**) Chromatin immunoprecipitation was conducted using an anti-H3k27me3 antibody in D1 cells that had been stimulated with curdlan for 1–4 h. (**C**) Chromatin immunoprecipitation was conducted using an anti-H3k4me3 antibody in D1 cells that had been stimulated with curdlan in the absence or presence of the NFAT inhibitor FK506. (**D**) Chromatin immunoprecipitation was conducted using an anti-H3k4me3 antibody in D1 cells that had been lentivirally transduced with a construct encoding NFATc2, or empty vector, before stimulation or not with curdlan for 2 h. (**E**) Luciferase reporter analysis. The luciferase reporter constructs containing the promoter of the indicated Zfp206 target genes were co-transfected into HEK293T somatic cells together with an NFATc2 expression construct or empty vector. (**F**) Measurement of mRNA levels in D1 cells by qRT-PCR. cDNA samples from D1 cells lentivirally transduced with a construct encoding NFATc2, or empty vector, before stimulation with curdlan for 2 h were interrogated with primers against IL2, IL12b and IL23a genes. Raw data were normalized to the housekeeping gene Actb and expression levels are presented relative to ActB. ChIP DNA was interrogated by qPCR using primers specific for IL2, IL12b and IL23 against polymerase primers. Fold-change values were normalized to the ChIP DNA detected in non-treated D1 cells. Data are presented as mean ± SD of triplicate experiments. *P < 0.01.

**Figure 6.**
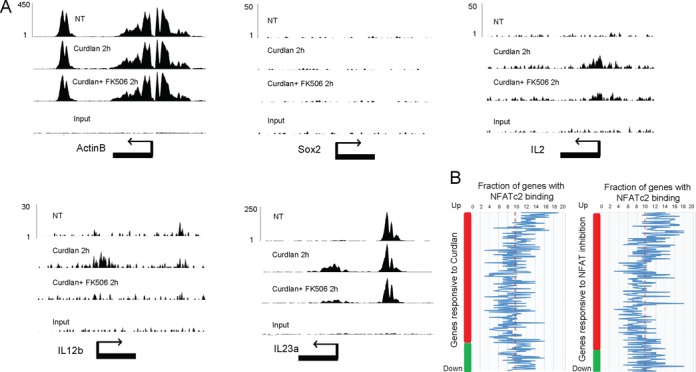
Genome-wide mapping of H3K4me3 in DCs treated with curdlan in the absence or presence of NFAT inhibitors. (**A**) NFATc2 promotes H3K4me3 modification of active genes but not silent genes in murine DCs. Examples of H3K4me3 loci at active cytokine genes IL2, IL12b and IL23a in DC stimulated or not with curdlan, either in the absence or presence of the NFAT inhibitor FK506. For comparison data from the silent non-cytokine gene Sox2 and the Actb housekeeping gene are presented alongside. Input DNA was used as control. (**B**) Preferential recruitment of NFATc2 to H3K4me3-modified target genes with upregulated expression. Genes with differential H3K4me4 marker expression fold change were identified by comparing ChIP-seq data from DCs treated or not with curdlan for 2 h, in the absence or presence of the NFAT inhibitor FK506. Moving average genes in a window of 50 genes that are within 20kb of NFATc2 ChIp-seq binding site are shown.

Specific histone modifications correlate with gene expression level, and H3K4me3 in particular is a well-established marker of active genes (47). Having demonstrated that NFATc2 binding functionally activates target genes, and given the rapid translocation and brief retention time of this transcription factor in the nucleus (Figure 1), we hypothesized that dectin-1 activation of DCs promotes NFATc2 translocation to the nucleus, where it binds to specific sequence motifs and recruits H3K4me3 to mediate chromatin opening and induce gene expression. To test this hypothesis, we performed ChIP-PCR to assess H3K4me3 occupancy of NFATc2 target genes at different time points after DC exposure to curdlan. We observed H3K4me3 marks in association with all NFATc2-regulated target genes within 2 h of curdlan stimulation, and H3K4me3 occupancy continued to increase over time (Figure 5A). However H3K27me3 occupancy was not affected (Figure 5B). The direct involvement of NFATc2 in the observed epigenetic modifications was evidenced by reduced H3K4me3 occupancy following treatment with the NFAT inhibitor FK506 (Figure 5C), and increased occupancy when NFATc2 was overexpressed (Figure 5D). The luciferase promoter reporter assay further confirmed the direct activation of NFATc2 to its target cytokine gene IL2, IL12b and Il23′s promoter (Figure 5E). In addition, overexpression of NFATc2 in DC led to significantly greater increases in gene expression of several target cytokines than in DC expressing normal levels of NFATc2 when both were stimulated with curdlan (Figure 5F). Taken together, NFATc2 is therefore required to mediate epigenetic modifications that drive cytokine and chemokine gene expression in activated DCs. Genome-wide mapping of H3K4me3 by ChIP-seq revealed NFAT-dependent enrichment of this mark at the IL2, IL12b and IL23a genes, but not at the non-cytokine gene Sox2 which is silent in DCs (Figure 6A). This enrichment was abrogated by exposure to the NFAT inhibitor FK506 (Figure 6A), clearly showing that NFATc2 is both specific and essential for mediating H3K4me3 enrichment at cytokine/chemokine genes in curdlan-activated DCs. Accordingly, we observed preferential association of NFATc2 with H3K4me3-modified genes that displayed upregulated expression in curdlan-stimulated DCs (Figure 6B), thus providing further evidence that NFATc2 plays a key role in epigenetic modification of gene activity in DCs.

### NFATc2 peak height and binding proximity to the TSS predicts gene expression level in DCs

**Figure 7.**
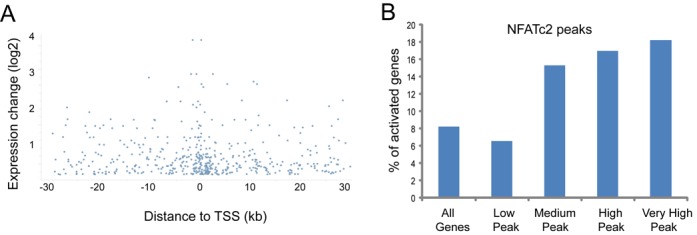
NFATc2 peak height and binding proximity to the TSS predict gene expression level in DCs. (**A**) Graph x-axis indicates the relative position of NFATc2 binding peaks relative to the TSS of the target genes, while the y-axis indicates the log_2_-fold change in gene expression in DCs after curdlan stimulation for 2 h, normalized to curdlan in the presence of NFAT inhibitor Fk506 for 2 h. (**B**) The peak height ranges were generated using Seqmonk software and were defined as low (0–20), medium (20–30), high (30–50) or very high (50–100).

It has been reported that the proximity of a transcription factor binding site to the TSS of a given gene can predict transcriptional activity (29), so we next assessed whether this applied to NFATc2 binding sites. When we plotted fold-change in expression of NFAT-regulated genes against the distance between the NFATc2 binding site and the TSS, we observed that the genes whose expression was most upregulated were those associated with an adjacent upstream NFATc2 binding site (Figure 7A). Dectin-1-activated genes displayed a similar proximity of NFATc2 binding sites to the TSS for the most upregulated genes, thus confirming that peak distance predicts the extent of gene activation, although we also observed a number of outliers that were perhaps regulated by other transcription factors such as NF-kB.

We next investigated whether NFATc2 ChIP peak height reflected the transcriptional activity of target genes. When we separated the ChIP peaks according to their height (low, medium, high or very high), and calculated the proportion of activated genes in each fraction, we observed that the percentage of activated genes for NFATc2 peaks of medium to very high height (15–18%) was at least 2-fold greater when compared to ‘low’ peaks (6%) (Figure 7B). These data demonstrated that NFATc2 peak height predicts the transcriptional activity of NFAT-regulated genes. Thus NFATc2 binding peak height and proximity to the TSS influence the level of cytokine and chemokine gene expression in dectin-1-activated DCs.

## DISCUSSION

In the current report we provide evidence that the transcription factor NFATc2 mediates epigenetic modification of DC cytokine responses to dectin-1 stimulation. Our data indicate that NFATc2 signaling is an essential mediator of myeloid cell responses to microbial antigens, consistent with previous reports that dectin-1 signaling in DCs is essential for effective host protection against fungal infections (4), and suggesting a broader role for NFAT signaling in immune regulation than that originally described in T cells more than two decades ago (14,48). Although it has long been established that ligation of TLRs/CLRs such as dectin-1 leads to rapid activation of calcineurin/NFAT signaling in DCs, until now the genetic targets of NFATc2 binding in these cells have remained obscure. Our data now reveal a critical role for NFATc2 signaling in regulating the DC response to microbial ligands, which is likely to influence the balance of inflammation versus regulation in infected host tissues.

We conducted genome-wide mapping of NFATc2 binding sites in dectin-1-stimulated DCs and integrated our findings with gene expression data to identify the directly regulated targets of this transcription factor. In addition to targets of NFATc2 such as the IL2 and Egr2/3 genes (2), we also detected NFATc2 regulation of the genes IL12b and IL23a that are required to produce the heterodimeric cytokine IL-23 and promote Th17 differentiation; we also observed a clear influence of NFATc2 on the transcription of genes encoding the growth factors GM-CSF and TGF-β3 that have previously been implicated in pathological Th17 differentiation (49–51). Similarly, TNFSF8 (CD153) has been reported to play critical roles in Th17 cell differentiation and proliferation (52), and our data revealed that NFATc2 directly regulates the expression of multiple TNF family members including TNFSF8, TNFSF14 and TNFSF18. Taken together, these data indicate that NFATc2 targets multiple cytokine genes that have been implicated in inflammatory pathologies across a variety of different tissues. In particular, the evidence of several targets in the Th17 pathway certainly warrants further investigation. We also observed that NFATc2 directly regulated a large group of chemokine genes with broad biological effects; CCL1 has been shown to influence regulatory T-cell function (53), CCL2 directs DCs to the tumor bed to promote killing of malignant cells (54), CCL3 contributes to the initiation of chronic myeloid leukemia (55) and CCL4 suppresses autoimmune destruction of islet β-cells (56). Furthermore, NFATc2 directly regulated the nur77 (Nr4a1) gene that promotes apoptosis of LPS-stimulated DCs in an NFATc2-dependent manner (21). Together, these data indicate that NFATc2 regulates a broad range of genes with diverse biological roles in DCs, including those involved in induction of Th17 differentiation, trafficking to peripheral tissues, and also DC survival, which together are likely to influence the outcome of inflammatory immune responses.

Among the thousands of NFATc2 binding targets identified here, only a small proportion conferred increased gene expression upon recruitment of NFATc2, which appears to be a common feature of transcription factor binding (29,38). Instead of exerting uniform effects on transcriptional activity, we observed that NFATc2 peak height and proximity to the TSS influenced the extent of gene activation, analogous to previous reports that have identified these same factors as key determinants of transcriptional repression (29). It is therefore possible that recruitment of additional co-factors to NFATc2 binding sites modifies the effects of this transcription factor on gene expression (38). Indeed, we found that the binding motifs of several well-known NFAT co-factors frequently flanked NFATc2 binding sites within directly regulated target genes, including AP1, JunB, JunD, PPAR and NFAT (Supplemental data).

An additional influence on gene expression levels is chromatin structure, which is remodeled by epigenetic modifications that control the access of transcriptional machinery to the template DNA. Chromatin remodeling can define different cell lineages, developmental stages and cellular responses to environmental signals (57), but until now NFAT's involvement has remained largely uncharacterized and no genome-wide roles for NFAT in epigenetic modification of gene expression had been reported. We observed a rapid shuttling of NFATc2 between cytoplasm and nucleus, preferential localization of NFATc2 to promoter sequences and H3K4me3 modification of multiple different target genes. In addition, we detected epigenetic modification of numerous different genes in dectin-1-activated DCs, including the cytokine genes IL2, IL12b and Il23a, which lacked H3K4me3 in the steady state but were significantly enriched in this mark following curdlan stimulation, except when in the presence of an NFAT inhibitor. Moreover, we detected preferential recruitment of NFATc2 to H3K4me3-enriched target genes on a genome-wide scale, thus confirming that NFATc2 regulates target gene expression by inducing specific epigenetic modifications.

Due to the established role of NFAT in regulating T-cell immunity, the calcineurin/NFAT inhibitors CsA and FK506 have been widely used to treat autoimmune diseases and suppress allograft rejection in human patients (58–60). Our study now demonstrates that the NFATc2 regulatory network also influences a broad range of DC functions and modulates the expression of multiple cytokines and chemokines that critically regulate host protection against bacteria and fungi. Improving our current understanding of the roles played by NFATc2-regulated genes in anti-microbial immune responses may therefore lead to the design of new drugs that more effectively target this pathway for the treatment of human inflammatory disorders and autoimmune pathologies.

## SUPPLEMENTARY DATA

Supplementary Data are available at NAR Online.

SUPPLEMENTARY DATA
