# Supplementary material for: NFATc2 mediates epigenetic modification of dendritic cell cytokine and chemokine responses to dectin-1 stimulation
Source: Nucleic Acids Res. 2014 Dec 30;43(2):836–47. doi: 10.1093/nar/gku1369 (PMC4333412; doi:10.1093/nar/gku1369)
Supplement: SUPPLEMENTARY DATA [file supp_43_2_836__index.html]

NFATc2 mediates epigenetic modification of dendritic cell cytokine and chemokine responses to dectin-1 stimulation — NFATc2 mediates epigenetic modification of dendritic cell cytokine and chemokine responses to dectin-1 stimulation — SUPPLEMENTARY DATA 

# NFATc2 mediates epigenetic modification of dendritic cell cytokine and chemokine responses to dectin-1 stimulation

## SUPPLEMENTARY DATA

**Files in this Data Supplement:**

- SUPPLEMENTARY DATA
- SUPPLEMENTARY DATA
- SUPPLEMENTARY DATA
- SUPPLEMENTARY DATA
