# Supplementary figures and images for: NFATc2 mediates epigenetic modification of dendritic cell cytokine and chemokine responses to dectin-1 stimulation
Source: Nucleic Acids Res. 2014 Dec 30;43(2):836–47. doi: 10.1093/nar/gku1369 (PMC4333412; doi:10.1093/nar/gku1369)

Figure S1

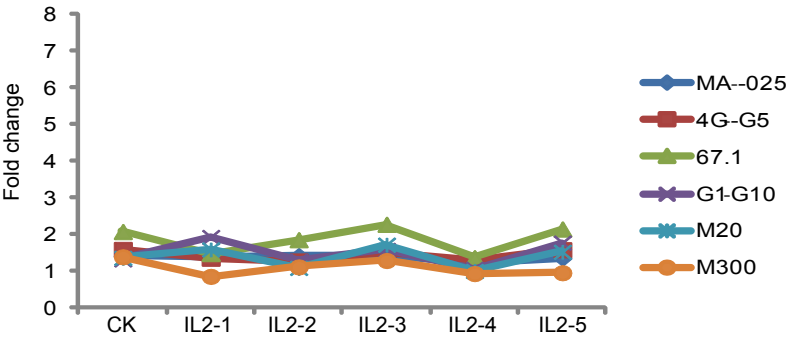

Figure S2

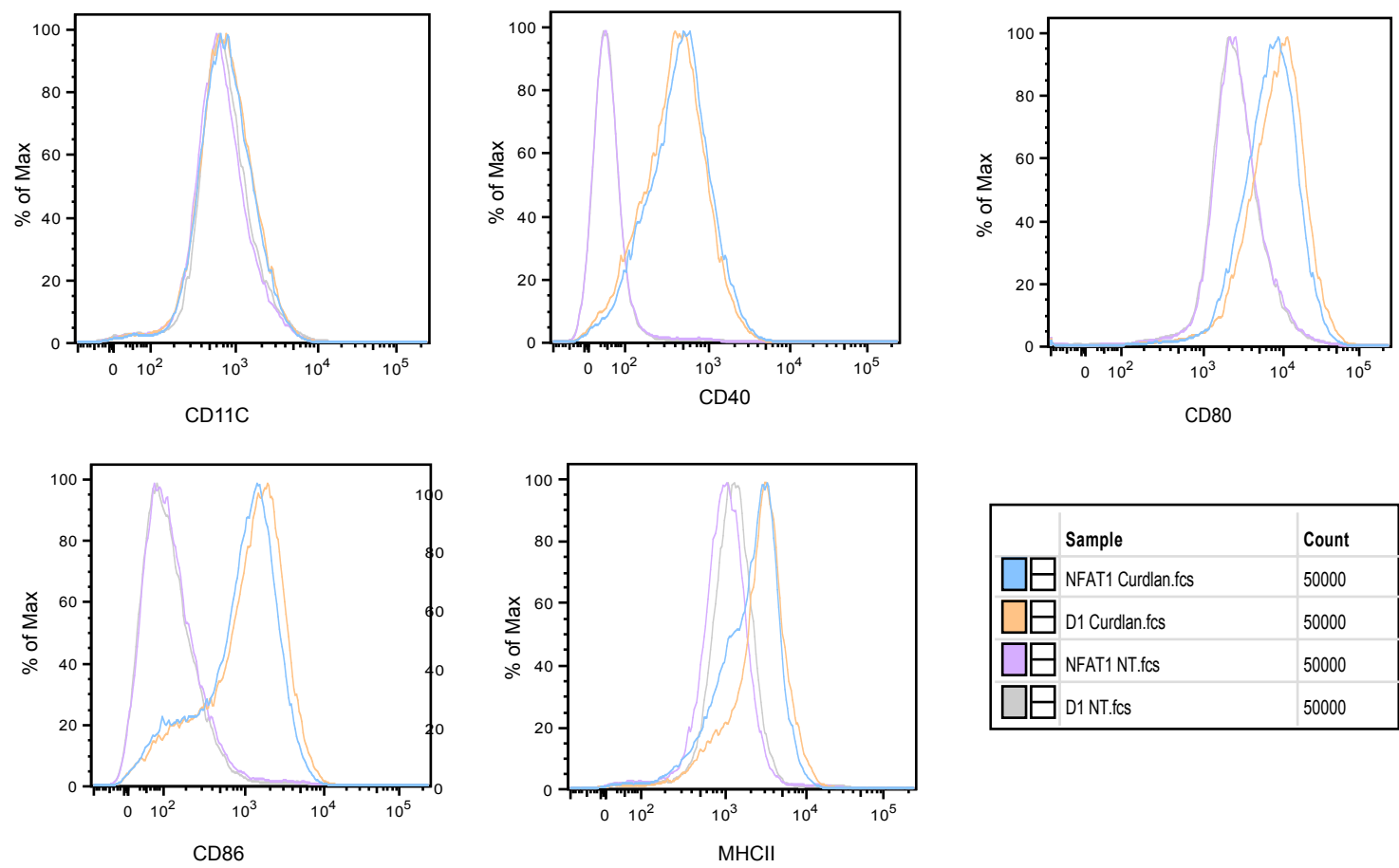

Figure S3

A

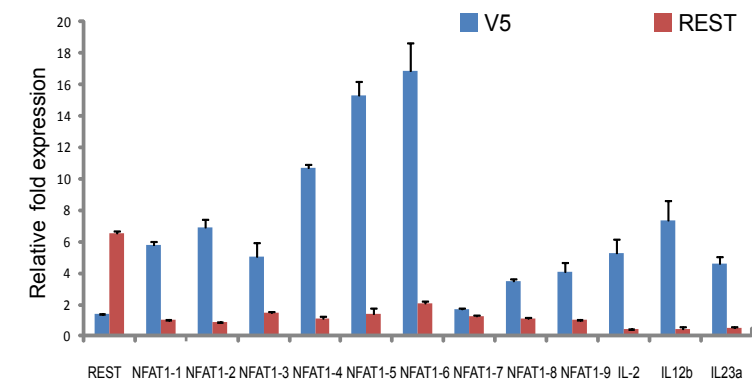

B

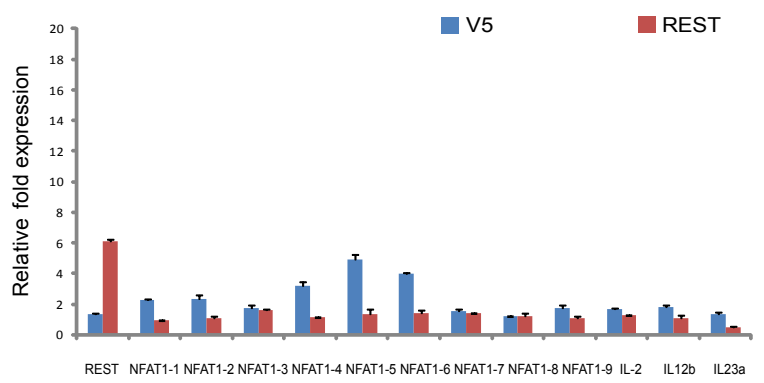

Figure S4

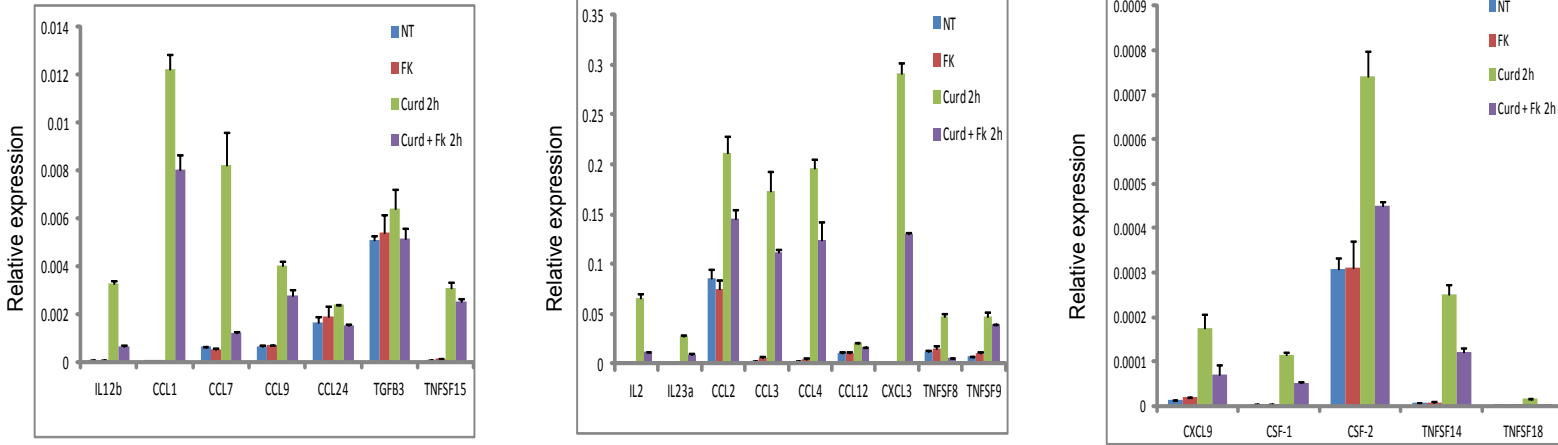

Supplement: SUPPLEMENTARY DATA [file supp_gku1369_nar-03496-z-2014-File004.pdf]
