# Supplementary material for: NFATc2 mediates epigenetic modification of dendritic cell cytokine and chemokine responses to dectin-1 stimulation
Source: Nucleic Acids Res. 2014 Dec 30;43(2):836–47. doi: 10.1093/nar/gku1369 (PMC4333412; doi:10.1093/nar/gku1369)
Supplement: SUPPLEMENTARY DATA [file supp_gku1369_nar-03496-z-2014-File003.docx]

**Table S1 NFATc2 binding cytokines and chemokine gene targets**

NFATc2 ChIP-seq Reads were mapped to the mouse genome (version mm9) with bowtie 1 using default parameters. The MACS 1.4.1 program was then used with default settings to detect peaks of ChIP enrichment ) relative to a control data set that was generated using input material from the same cell line. The binding targets were allocated to the nearest gene targets. From the whole list, the cytokines and chemokines were listed as in table..

| **Cytokines** | | **Chemokines** | |
| --- | --- | --- | --- |
| Gene accession | Gene name | Gene accession | Gene Name |
| NM_008366.3 | IL2 | NM_011329.3 | Ccl1 |
| NM**_**031252.2 | IL23a | NM_011333.3 | Ccl2 |
| NM_008352.2 | IL12b | NM_011337.2 | Ccl3 |
| NM_145837.3 | IL17d | NM_013652.2 | Ccl4 |
| NM_010556.4 | IL3 | NM_013653.3 | Ccl5 |
| NM_009969.4 | Csf2 | NM_009139.3 | Ccl6 |
| NM_010548.2 | IL10 | NM_013654.3 | Ccl7 |
| NM_016971.2 | IL22 | NM_021443.3 | Ccl8 |
| NM_010554.4 | IL1a | NM_011338.2 | Ccl9 |
| NM_008361.3 | IL1b | NM_011330.3 | Ccl11 |
| NM_011329.3 | Ccl1 | NM_011331.2 | Ccl12 |
| NM_010118.3 | Egr2 | NM_011332.3 | Ccl17 |
| NM_001289925.1 | Egr3 | NM_019577.4 | Ccl24 |
| NM_009452.2 | Tnfsf4 | NM_009140.2 | Cxcl2 |
| NM_009403.3 | Tnfsf8 | NM_203320.3 | Cxcl3 |
| NM_009404.3 | Tnfsf9 | NM_009141.3 | Cxcl5 |
| NM_019418.3 | Tnfsf14 | NM_008599.4 | Cxcl9 |
| NM_177371.3 | Tnfsf15 | NM_018866.2 | Cxcl13 |
| NM_183391.3 | Tnfsf18 | NM_019568.2 | Cxcl14 |
| NM_001278601.1 | Tnf | NM_023158.6 | Cxcl16 |
| NM_009368.3 | Tgfb3 |  |  |

**Table S2 Differentially expressed genes identified by microarray ***

The Affymetrix Mouse Gene 1.0ST Arrays were used to profile in curdlan-stimulated D1 cells (2h or 4h exposure) that had been treated or not with FK506 for the duration of culture. A Benjamini-Hochberg-corrected P-value <0.05 and cut-off fold-change value of ≥1.5 were applied to identify genes that displayed statistically significant changes in expression.

|  | Curdlan/NT  2h | Curdlan/NT  4h | Curdlan/Curdlan+FK  2h | Curdlan/Curdlan+FK  4h |
| --- | --- | --- | --- | --- |
| Up | 515 | 1245 | 171 | 380 |
| Down | 323 | 896 | 82 | 41 |

*****fold change>1.5, P-value>0.05

**Table S3 Cytokines and chemokines directly regulated by NFATc2**

The NFATc2 ChIP-seq and microarray data were integrated to generate NFATc2 direct regulated targets.. The cytokines and chemokines that were induced upon curdlan stimulation but were repressed with NFAT inhibitor FK506 by microarray data, and also contain an NFATc2 binding site identified by NFATc2 ChIP-seq, were listed as NFATc2 direct regulated cytokines and chemokines.

| **Cytokines** | | | **Chemokines** | | |
| --- | --- | --- | --- | --- | --- |
| Gene  Name | Curd 2h | Curdlan/  FK506 2h | Gene  Name | Curd 2h | Curdlan/  FK506 2h |
|  | Fold change | Fold change |  | Fold change | Fold change |
| IL2 | 77.5 | 3.0 | Ccl1 | 42.2 | 3.2 |
| IL12b | 9.3 | 4.1 | Ccl2 | 2.1 | 2.1 |
| IL23a | 26.4 | 2.1 | Ccl3 | 5.6 | 1.5 |
| Tnfsf8 | 2.9 | 3.3 | Ccl4 | 28.6 | 5.9 |
| Tnfsf9 | 5.8 | 1.4 | Ccl7 | 16.7 | 13.4 |
| Tnfsf14 | 6.5 | 6.2 | Ccl9 | 4.4 | 2.7 |
| Tnfsf15 | 6.9 | 2.4 | Ccl12 | 2.9 | 1.8 |
| Tnfsf18 | 1.9 | 2.1 | Ccl24 | 1.7 | 1.6 |
| Csf1 | 11.2 | 2.6 | Cxcl3 | 1.5 | 1.4 |
| Csf2 | 4.2 | 3.2 | Cxcl9 | 2.2 | 2.0 |
| Tgfb3 | 1.4 | 1.5 |  |  |  |

**Supplemental Figure Legends**

**Figure S1. NFAT1 binding at the IL2 promoter.** D1 cells were stimulated with curdlan for 30min before being subjected to chromatin immunoprecipitation using a commercially available antibody against NFAT1. Primers flanking the IL2 promoter ~2.5kb upstream of the TSS were used. Data are presented as mean ± s.d. of duplicate experiments.

**Figure S2. Maturation of NFAT1-tagged D1 cells.** NFAT1-tagged D1 cells and control D1 cells were treated with 10µg curdlan for 18h and then analysed by flow cytometry using antibodies against CD11c, CD40, CD80, CD86 and MHCII.

**Figure S3. Validation of NFAT1 binding sites using ChIP-PCR. A** and **B.** Chromatin immunoprecipitation of V5-tagged NFAT1 proteins was conducted using an anti-V5 antibody (or anti-REST control) in NFAT1-tagged D1 cells that had been stimulated with curdlan for 30min either in the absence (A) or presence (B) of the NFAT inhibitor FK506. ChIP DNA was interrogated by qPCR using 9 randomly selected primers as positive controls and REST primers as negative controls. Data are presented as mean ± s.d. of duplicate experiments.

**Figure S4. Validation of differentially expressed cytokine genes identified by microarray.** Quantitative PCR analysis of cytokine mRNA levels in D1 cells after treatment with FK506 and curdlan, either alone or in combination, for comparison with non-treated control cells (NT). Samples of cDNA were prepared and then interrogated using specific primers for the house keeping gene Actb and various cytokine genes. Data are presented as mean ± s.d. of duplicate experiments.
